# Characterization and expression analysis of a newly identified glutathione S-transferase of the hard tick *Haemaphysalis longicornis* during blood-feeding

**DOI:** 10.1186/s13071-018-2667-1

**Published:** 2018-02-08

**Authors:** Emmanuel Pacia Hernandez, Kodai Kusakisako, Melbourne Rio Talactac, Remil Linggatong Galay, Takeshi Hatta, Tomohide Matsuo, Kozo Fujisaki, Naotoshi Tsuji, Tetsuya Tanaka

**Affiliations:** 1 Laboratory of Infectious Diseases, Joint Faculty of Veterinary Medicine, Kagoshima University, 1-21-24 Korimoto, Kagoshima, 890-0056 Japan; 2 Department of Pathological and Preventive Veterinary Science, The United Graduate School of Veterinary Science, Yamaguchi University, Yoshida, Yamaguchi, 753-8515 Japan; 3 Department of Clinical and Population Health, College of Veterinary Medicine and Biomedical Sciences, Cavite State University, 4122 Cavite, Philippines; 4 Department of Veterinary Paraclinical Sciences, University of the Philippines at Los Baños, College, 3004 Laguna, Philippines; 5 Department of Parasitology, Kitasato University School of Medicine, Kitasato, Minami, Sagamihara, Kanagawa 252-0374 Japan; 6 Laboratory of Parasitology, Joint Faculty of Veterinary Medicine, Kagoshima University, 1-21-24 Korimoto, Kagoshima, 890-0056 Japan; 7 National Agricultural and Food Research Organization, 3-1-5 Kannondai, Tsukuba, Ibaraki 305-0856 Japan

**Keywords:** Glutathione S-transferases, *Haemaphysalis longicornis*, Tick, Oxidative stress, Blood-feeding

## Abstract

**Background:**

Ticks are obligate hematophagous parasites important economically and to health. Ticks consume large amounts of blood for their survival and reproduction; however, large amounts of iron in blood could lead to oxidative stress. Ticks use several molecules such as glutathione S-transferases (GSTs), ferritins, and peroxiredoxins to cope with oxidative stress. This study aimed to identify and characterize the GSTs of the hard tick *Haemaphysalis longicornis* in order to determine if they have a role in coping with oxidative stress.

**Methods:**

Genes encoding GSTs of *H. longicornis* were isolated from the midgut CDNA library. Genes have been cloned and recombinant GSTs have been expressed. The enzymatic activities, enzyme kinetic constants, and optimal pH of the recombinant GSTs toward 1-chloro-2,4-dinitrobenzene (CDNB) were determined. The gene transcription and protein expression profiles were determined in the whole ticks and internal organs, and developmental stages using real time RT-PCR and Western blotting during blood feeding. The localization of GST proteins in organs was also observed using immunofluorescent antibody test (IFAT).

**Results:**

We have isolated two genes encoding GSTs (*HlGST* and *HlGST*2). The enzymatic activity toward CDNB is 9.75 ± 3.04 units/mg protein for recombinant HlGST and 11.63 ± 4.08 units/mg protein for recombinant HlGST2. Kinetic analysis of recombinant HlGST showed *K*_*m*_ values of 0.82 ± 0.14 mM and 0.64 ± 0.32 mM for the function of CDNB and GSH, respectively. Meanwhile, recombinant HlGST2 has *K*_*m*_ values of 0.61 ± 0.20 mM and 0.53 ± 0.02 mM for the function of CDNB and GSH, respectively. The optimum pH of recombinant HlGST and recombinant HlGST2 activity was 7.5–8.0. Transcription of both *GST*s increases in different developmental stages and organs during blood-feeding. GST proteins are upregulated during blood-feeding but decreased upon engorgement in whole ticks and in some organs, such as the midgut and hemocytes. Interestingly, salivary glands, ovaries, and fat bodies showed decreasing protein expression during blood-feeding to engorgement. Varying localization of GSTs in the midgut, salivary glands, fat bodies, ovaries, and hemocytes was observed depending on the feeding state, especially in the midgut and salivary glands.

**Conclusions:**

In summary, a novel GST of *H. longicornis* has been identified*.* Characterization of the GSTs showed that GSTs have positive correlation with the degree and localization of oxidative stress during blood-feeding. This could indicate their protective role during oxidative stress.

**Electronic supplementary material:**

The online version of this article (10.1186/s13071-018-2667-1) contains supplementary material, which is available to authorized users.

## Background

Ticks are obligate hematophagous parasites prevalent worldwide. They serve as several disease vectors in humans and other animals [1]. *Haemaphysalis longicornis* is a tick with a distribution in Australia, New Zealand and eastern Asia [2]. Ticks are known for their ability to ingest large volumes of blood from their hosts [3]. Blood contains potentially toxic molecules, such as iron, which can promote the production of hydroxyl radicals and reactive oxygen species (ROS) that can lead to oxidative stress [4]. Therefore, ticks must have protective mechanisms against oxidative stress. Previous studies have shown the role of ferritins, catalases and peroxiredoxins as coping mechanism during periods of oxidative stress [5].

Glutathione S-transferases (GSTs) are enzymes known to conjugate xenobiotic compounds, such as drugs and pesticides, with glutathione (GSH) for their metabolism. Aside from this, they are also involved in the catalysis of fatty acid reduction and the metabolism of phospholipids and DNA hydroperoxidases, which are all products of oxidative stress [6]. Several studies of GSTs either involved measuring their enzymatic activity [7] or analyzing their gene expression profile [8]; however, these methods have limitations. According to Hayes et al. [9], studies of GST activity do not take into consideration GST’s ability to bind to molecules other than its substrate that would inhibit its activity. On the other hand, several studies have shown the presence of post transcriptional factors that could present differences between gene and protein expressions of GSTs [10, 11]. Thus, studies involving immunohistochemistry are necessary to establish the relationship between GST localization and its function to fully understand the tick detoxification pathway involving GSTs.

A GST of *H. longicornis* has been previously identified and partially characterized [2]. Additionally, GSTs of other tick species such as *Rhipicephalus* (*Boophilus*) *microplus*, *R. appendiculatus*, *Dermacentor variabilis*, *R.* (*B.*) *annulatus* and *R. sanguineus*, have been identified and characterized [2, 12–16]. Here we have identified a novel GST from *H. longicornis* and characterized its role through its activity, gene transcription, protein expression, and protein localization during the course of blood-feeding to be able to evaluate its potential in designing a new method of tick control.

## Methods

### Ticks and experimental animals

The parthenogenetic Okayama strain of *H. longicornis* was used in all experiments throughout this study. Ticks were maintained by feeding on the ears of Japanese white rabbits (KBT Oriental, Saga, Japan) for several generations at the Laboratory of Infectious Diseases, Joint Faculty of Veterinary Medicine, Kagoshima University, Kagoshima, Japan [17]. Rabbits were also used in all tick infestation experiments. Twelve 4-week-old female ddY mice (Kyudo, Kumamoto, Japan) were used for GST antiserum preparation. Experimental animals were kept at 25 °C and 40% relative humidity, with a constant supply of water and commercial feeds. The ticks, on the other hand was maintained in glass tubes sealed with cotton plug and maintained at 15 °C and 80–85% relative humidity in an incubator until use. The care and use of experimental animals in this study were approved by the Animal Care and Use Committee of Kagoshima University (approval numbers VM15055 and VM15056 for the rabbits and mice, respectively).

### Identification and characterization of *GST* cDNA clones

The expressed sequence tags (EST) database of *H. longicornis* was analyzed and searched for genes encoding GSTs. Plasmids containing inserts for the two *GST*s were extracted using the Qiagen Plasmid Mini Kit (Qiagen, Hilden, Germany) and underwent sequencing using an automated sequencer (ABI PRISM 3100 Genetic Analyzer; Applied Biosystems, Foster City, CA, USA) to determine the full-length sequence. The deduced amino acid translation of GST genes was determined using GENETYX software (Genetyx, Tokyo, Japan). A homologous search of the full-length *GST* sequences was performed using BLAST programs, through which conserved domains were also identified. The presence of a signal peptide was checked using the SignalP 3.0 prediction server (http://www.cbs.dtu.dk/services/SignalP/), and the predicted molecular weight and isoelectric points (pIs) were determined using the ExPASy server (http://web.expasy.org/peptide_mass/). Analysis for *N*-glycosylation was performed using the NetNGLyc 1.0 server (http://www.cbs.dtu.dk/services/NetNGlyc/). A phylogenetic tree was constructed based on the amino acid sequences of GSTs from selected species by the neighbor-joining method using the Phylogeny.fr server (http://www.phylogeny.fr/). Multiple sequence alignments between GSTs among tick species were also done using the BOXSHADE software (http://www.ch.embnet.org/software/BOX_form.html). Molecular models of GSTs were also constructed using PHYRE2 software (http://www.sbg.bio.ic.ac.uk/phyre2/) and analyzed using PyMOL software (www.pymol.org).

### Preparation of recombinant GSTs

The open reading frames (ORF) of the two *GST* genes were amplified using gene-specific primers: HlGST *Bam*HI forward, HlGST *Eco*RI reverse, HlGST2 *Bam*HI forward, and HlGST2 *Eco*RI reverse (Table 1). PCR was conducted using a KOD-Plus-Neo PCR Kit (Toyobo, Osaka, Japan) following the manufacturer’s protocol. The PCR profile was as follows: 94 °C for 2 min, 45 cycles of the denaturation step at 98 °C for 10 s, and an annealing/extension step at 68 °C for 45 s. PCR products were purified using a GENECLEAN II Kit (MP Biomedicals, Solon, OH, USA), and then subcloned into the pRSET A vector (Invitrogen, Carlsbad, CA, USA). The resulting plasmids were checked for accurate insertion through the analysis by restriction enzymes *Bam*HI and *Eco*RI, and the target sequences were read using the automated sequencer. The plasmids were purified using the Qiagen Plasmid Mini Kit (Qiagen). The purified plasmids were expressed in *Escherichia coli* BL21 cells, grown in Luria-Bertani (LB) broth medium with ampicillin. The synthesis of recombinant GSTs tagged with histidine was induced with isopropyl β-D-1-thiogalactiopyranoside (IPTG) at a final concentration of 1 mM. Cells were collected by centrifugation, and protein was extracted through ultrasonication. Purification was carried out using a HisTrap column (GE Healthcare, Uppsala, Sweden) and then dialyzed against phosphate buffered saline (PBS). The purity was checked using sodium dodecyl sulphate polyacrylamide gel electrophoresis (SDS-PAGE) analysis and Western blotting using the anti-Histidine antibody (GE Healthcare). The protein concentration was determined through SDS-PAGE using bovine serum albumin as the standard. Micro BCA Protein Assay Kit (Thermo Scientific, Rockford, IL, USA) was also used to check the protein concentration.

**Table 1:** Gene-specific primers used in this study. Underlined letters indicate enzyme recognition sites. RNA polymerase promoter sequence is indicated in italic

Primer	Sequence (5′ → 3′)
HlGST real-time forward	CTTCTTGGATCTTGGCGGGT
HlGST real-time reverse	CGATGTCCCAGTAGCCGAG
HlGST *Bam*HI forward	CGGGATCCATGGCTCCTATTCTCGGCT
HlGST *Eco*RI reverse	CGGAATTCTCAGCAGTCGTCAGCGGGCG
HlGST RT forward	ACGTGAAGCTCACCCAGAGCAT
HlGST RT reverse	AAGCTAGCCATGTCGCCGTTGA
HlGST RNAi forward	GCCTGGCTCAAGGAGAAACACA
HlGST RNAi reverse	ACAAAGGCCTTCAGGTTGGGGA
HlGST T7 forward	*TAATACGACTCACTATAGG*GCCTGGCTCAAGGAGAAACACA
HlGST T7 reverse	*TAATACGACTCACTATAGG*ACAAAGGCCTTCAGGTTGGGGA
HlGST2 real-time forward	CCCTTCCGGGAATGAAGGAG
HlGST2 real-time reverse	GATCGCTCAGCAGTCGTCAG
HlGST2 *Bam*HI forward	CGGGATCCATGGCCCCTGTGCTGGGATA
HlGST2 *Eco*RI reverse	CGGAATTCTCAGCAGTCGTCAGCGGGCG
HlGST2 RT forward	ACGTCAAGCTGACGCAGAGCAT
HlGST2 RT reverse	ATGGGCCAAGCCTTGAAGCGAT
HlGST2 RNAi forward	AGGATAAAAGGTACGGCTTCGGCA
HlGST2 RNAi reverse	TTTCACGATCTGGAGAGCCTCGTA
HlGST2 T7 forward	*TAATACGACTCACTATAGG*AGGATAAAAGGTACGGCTTCGGCA
HlGST2 T7 reverse	*TAATACGACTCACTATAGG*TTTCACGATCTGGAGAGCCTCGTA
P0 real-time forward	CTCCATTGTCAACGGTCTCA
P0 real-time reverse	TCAGCCTCCTTGAAGGTGAT
L23 real-time forward	CACACTCGTGTTCATCGTCC
L23 real-time reverse	ATGAGTGTGTTCACGTTGGC
Actin real-time forward	ATCCTGCGTCTCGACTTGG
Actin real-time reverse	GCCGTGGTGGTGAAAGAGTAG
Actin RT forward	CCAACAGGGAGAAGATGACG
Actin RT reverse	ACAGGTCCTTACGGATGTCC
Tubulin real-time forward	TTCAGGGGCCGTATGAGTAT
Tubulin real-time reverse	TGTTGCAGACATCTTGAGGC
EGFP T7 forward	*TAATACGACTCACTATAGG*GACGTAAACGGCCACAAGTT
EGFP T7 reverse	*TAATACGACTCACTATAGG*TGCTCAGGTAGTGGTTGTCG

### Enzyme activity assay

The enzymatic activity of recombinant GSTs was measured according to the methods of Habig [18] using 1-chloro-2,4-dinitrobenzene (CDNB) (Sigma-Aldrich, St. Louis, MO, USA) as a substrate. Two hundred microliters of the reaction mixture consisting of a final concentration of 1 mM CDNB dissolved in methanol, 5 mM glutathione, and 120 μM recombinant GSTs in 100 mM Tris-HCl (pH 7.5) or without recombinant GST for the blank was tested in a 96-well plate. Methanol concentration was maintained at 5%. Equine liver GST and the *H. longicornis* peroxiredoxin enzyme were used as the positive and negative control, respectively. The absorbance (A_340nm_) was measured each minute in an SH-9000 microplate reader (Corona Electric, Ibaraki, Japan) at 25 °C for 5 min. The extinction coefficient of 9.6 mM^-1^cm^-1^, corrected for the 96-well microplate light path, was used. Each assay was done in triplicate, and the results were expressed as the mean of three separate experiments. The effect of pH on the recombinant GSTs was measured, using the previously described procedure and changing the buffer to either 100 mM citrate buffer (pH 5.0 and 5.5) or Tris-HCl buffer (pH 6.8, 7.5, 8.0 and 9.5).

The same procedure was utilized for enzymatic activity; however, different concentrations of CDNB (0.125, 0.25, 0.5, 1 and 2 mM) in methanol and a constant 5 mM GSH or different concentrations of GSH (0.5, 1, 2 and 5 mM) with a constant 1 mM CDNB in 100 mM Tris-HCl buffer (pH 7.5) were used. Each assay was done in triplicate, and the results are expressed as the mean of three separate experiments. Kinetic constants *K*_*m*_ and *V*_*max*_ were calculated from a double-reciprocal plot of 1/v *versus* a 1/[S] or Lineweaver-Burk plot in which *V*_*max*_ = 1/y-intercept of the regression line and *K*_*m*_ = *V*_*max*_ × slope of the regression line.

### Preparation of mouse anti-GST sera

To prepare mouse anti-GST sera, 6 mice for each GST were used and each mouse was injected intraperitoneally with 0.5 ml of 200 μg/ml of recombinant GST completely mixed with an equal volume of Freund’s Complete Adjuvant (Sigma-Aldrich) to give each mouse 100 μg recombinant protein. Immunization was repeated 14 and 28 days after the first immunization; however, recombinant GST was mixed with incomplete adjuvant (Sigma-Aldrich). All sera were collected 14 days after the last immunization. Antisera were tested using Western blotting, using both recombinant GSTs and tick protein.

### RNA interference

RNA interference using double-stranded RNA (dsRNA) was performed to check for cross-reactivity between the GST antisera. The PCR primers used for the synthesis of dsRNA are listed in Table 1. The *HlGST* and *HlGST*2 fragments were amplified by PCR from plasmid clones using oligonucleotides, including HlGST T7 forward with HlGST RNAi reverse and HlGST T7 reverse with HlGST RNAi forward primers, as well as HlGST2 T7 forward with HlGST2 RNAi reverse and HlGST2 T7 reverse with HlGST2 RNAi forward primers, to attach the T7 promoter recognition sites on both forward and reverse ends. Enhanced green fluorescent protein (*EGFP*) was amplified from *pEGFP* through PCR using oligonucleotides containing EGFP T7 forward and EGFP T7 reverse primers as well. PCR products were purified using a GENECLEAN II Kit (MP Biomedicals). The T7 RiboMAX Express RNAi System (Promega, Madison, WI, USA) was used to synthesize dsRNA by in vitro transcription. The successful construction of dsRNA was confirmed by running 1 μl of the dsRNA products in 1.5% agarose gel in a TAE buffer. 0.5 μl of 2 μg/μl of *HlGST*, *HlGST2*, and *HlGST1/2* (*HlGST* and *HlGST2* mixed at 2 μg/μl concentration each) dsRNA dissolved in high-purity water were injected to the hemocoel of unfed adult female ticks through the fourth coxae to give each tick 1 μg of dsRNA. A total of 10 ticks per group were injected with dsRNA. The control group was injected with *EGFP* dsRNA. After injection, the ticks were held for 24 h in a 25 °C incubator to check for mortality resulting from injury during injection. The ticks were then to feed on rabbits for four days, and then the partially fed ticks were collected. Total RNA was extracted from five whole 4-day-fed ticks, and their cDNA was synthesized. cDNA was subjected to RT-PCR with a Hot Start Pol system (Jena Bioscience, Jena, Germany) using *GST-*specific primers, HlGST RT forward and HlGST RT reverse primers, and HlGST2 RT forward and HlGST2 RT reverse primers, following the manufacturer’s instructions. The PCR cycle profile was as follows: 94 °C for 8 min, 30 cycles of a denaturation step at 94 °C for 30 s, an annealing step at 68 °C for 60 s, and an extension step at 72 °C for 60 s. The PCR products were run in 1.5% TAE agarose gels and stained with ethidium bromide. *Actin* was used as a loading control. The absence of bands corresponding to *HlGST* and *HlGST2* in their corresponding GST knockdown group demonstrates that silencing was successful (Additional file 1: Figure S1). Proteins were extracted and prepared from the remaining ticks of different knockdown groups for Western blotting using the prepared anti-GST sera. Mouse tubulin antiserum was used as a control for Western blotting. The absence of signals corresponding to HlGST in the *HlGST* knockdown group and HlGST2 in the *HlGST2* knockdown group demonstrates that the antibodies produced are specific and do not cross react; therefore, they could be used in succeeding experiments (Additional file 1: Figure S1).

### Total RNA extraction and real-time PCR analysis

Total RNA was extracted from different developmental stages (egg, larva, nymph and adult) and organs of adult female ticks, including the midgut, salivary glands, ovaries, fat body, and hemocytes during blood-feeding. Whole tick samples were homogenized using an automill (Tokken, Chiba, Japan) and were added to TRI Reagent® (Sigma-Aldrich). On the other hand, organs such as salivary glands, midguts, fat bodies, and ovaries were dissected and washed in PBS, placed directly in tubes with the TRI Reagent, and homogenized. Hemocytes were collected through the legs of ticks as previously described [19]. RNA extraction was performed following the manufacturer’s protocol. Subsequently, single-strand cDNA was prepared by reverse transcription using the ReverTra Ace® cDNA Synthesis Kit (Toyobo), following the manufacturer’s protocol. Transcription analysis of *HlGST* and *HlGST2* genes was performed through real-time PCR using THUNDERBIRD™ SYBR® qPCR Mix (Toyobo) with an Applied Biosystems 7300 Real-Time PCR System using gene-specific primers, HlGST real-time forward and HlGST real-time reverse, and HlGST2 real-time forward and HlGST2 real-time reverse primers (Table 1). Standard curves were made from fourfold serial dilutions of the cDNA of adult ticks fed for 3 days. The PCR cycle profile was as follows: 95 °C for 10 min, 40 cycles of a denaturation step at 95 °C for 15 s, and an annealing/extension step at 60 °C for 60 s. The data was analyzed with Applied Biosystems 7300 system SDS software. In the first step of real-time PCR, *actin*, *tubulin*, *P0*, and *L23* genes were evaluated for standardization. *P0* genes were selected as an internal control for the whole ticks, while *L23* genes were chosen for the tick organs.

### Protein extraction and Western blotting analysis

Protein was extracted at different developmental stages and from different organs of adult female ticks during blood-feeding. For different developmental stages, whole tick samples were homogenized using an automill (Tokken), and then suspended in PBS treated with Complete Mini Proteinase Inhibitor Cocktail Tablets (Roche, Mannheim, Germany). Eggs and organs were homogenized using a mortar, and they were also suspended in PBS treated with a proteinase inhibitor. After sonication and recovery of the supernatant, tick proteins were separated with a 12% SDS-polyacrylamide gel electrophoresis (SDS-PAGE) and transferred to a polyvinylidene difluoride (PVDF) membrane (Millipore, Billerica, MA, USA). The membrane was blocked overnight with 3% skim milk in PBS with 0.05% Tween 20, and then incubated with a primary antibody using mouse anti-GST sera (1:1000 dilution) for 1 h. β-tubulin was used as a control [20]. After incubation with horseradish peroxidase-conjugated goat anti-mouse IgG (1:50,000 dilution; DakoCytomation, Glostrup, Denmark) for 1 h, the signal was detected using Clarity™ Western ECL Substrate (Bio-Rad Laboratories, Hercules, CA, USA) or Amersham™ ECL™ Prime Western Blotting Detection Reagent (GE Healthcare, Buckinghamshire, UK). It was analyzed using FluorChem FC2 software (Alpha Innotech, San Leandro, CA, USA).

### Indirect immunofluorescent antibody test

An indirect immunofluorescent antibody test (IFAT) was performed to demonstrate the endogenous localization of GSTs, as described previously [21]. The salivary glands, midguts, ovaries, and fat bodies of ticks at different stages of blood-feeding were immediately dissected under a stereo microscope. Dissected organs were fixed overnight in 4% paraformaldehyde in PBS with 0.1% glutaraldehyde and then washed with different concentrations of sucrose in PBS. The organs were embedded in Tissue-Tek OCT Compound (Sakura Finetek Japan, Tokyo, Japan) and then frozen using liquid nitrogen. Sections 10 μm thick were cut using a cryostat (Leica CM3050; Leica Microsystems, Wetzlar, Germany) and placed on MAS-coated glass slides (Matsunami Glass, Osaka, Japan). After blocking for 1 h with 5% skim milk in PBS at room temperature, sections were incubated with a 1:100 dilution of anti-GST sera overnight at 4 °C. Normal mouse serum at the same dilution was used as a negative control. Sections were washed with PBS and then incubated with Alexa Fluor 488-conjugated goat anti-mouse IgG (1: 1000; Invitrogen) for 1 h at room temperature. After washing with PBS, sections were mounted in Vectashield with DAPI (Vector Laboratories, Burlingame, CA, USA). Images were taken using a confocal fluorescence microscope mounted with an LSM 700 (Carl Zeiss, Jena, Germany).

### Statistical analysis

Student’s t-test was used to analyze data from the real-time PCR of ticks and organs. A significant difference is defined as *P* < 0.05. All experiments were done at least twice for validation.

## Results

### Identification and characterization of *GST* cDNAs

**Fig. 1:**
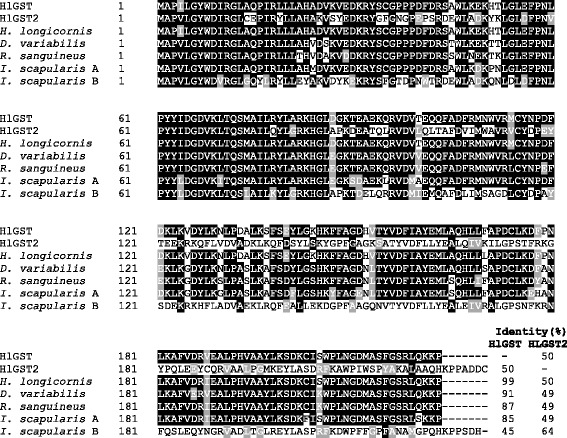
Multiple sequence alignment of the deduced amino acid sequences of HlGST and HlGST2 with other tick GSTs. Identical residues are shaded black, while similar residues are shaded gray. The percent identities with HlGST and HlGST2 are placed at the end of the sequences. The GenBank accession numbers for GST sequences are as follows: *Haemaphysalis longicornis* (AAQ74441.1), *Dermacentor variabilis* (ACF35504.1), *Rhipicephalus sanguineus* (AGK29895.1), *Ixodes scapularis* A (XP_002401749.1) and *Ixodes scapularis* B (XP_002434207.1)

Two cDNAs encoding glutathione S-transferase were identified and cloned. The open reading frame of the first *GST* gene contains 672 base pairs (bp) from the predicted start codon to the predicted end codon, encodes 223 amino acid polypeptides, and has a calculated molecular weight of ~25.7 kDa and a pI of 7.67. No glycosylation site or signal peptide was predicted. An N-terminal domain containing the glutathione binding site and a C-terminal domain containing the 105 amino acids substrate binding site were present. Since BLAST and multiple sequence alignment analysis showed that it has 99% homology with the previously identified *H. longicornis* GST (HlGST) (GenBank: AAQ74441), it is considered the same as HlGST (Additional files 2 and 3: Figures S2 and S3). It also showed high homology to the GSTs of *D. variabilis* (91%), *R. sanguineus* (87%), and *I. scapularis* (GenBank: XP_002401749.1) (85%) (Fig. 1).

On the other hand, the open reading frame of the second GST has 693 bp from the predicted start codon to the predicted end codon, encodes 230 amino acid polypeptides, and has a calculated molecular weight of ~26.3 kDa and a pI of 6.83. No glycosylation site or signal peptide was predicted. It also has an N-terminal domain containing the glutathione binding site and a C-terminal domain containing the 65 amino acids substrate binding site. (Additional files 2 and 3: Figures S2 and S3). We found the second GST novel and submitted to GenBank where it was assigned the accession number LC169599. We refer to the novel GST as HlGST2. HlGST2 has 64% homology with the putative GST of *I. scapularis* (GenBank: XP_002434207.1) (Fig. 1).

**Fig. 2:**
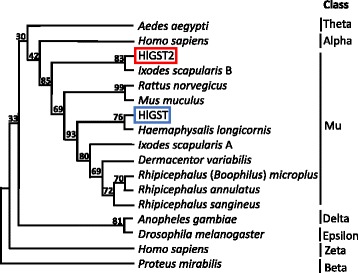
Phylogenetic tree of GSTs from different species of ticks, selected vertebrates, and invertebrates. A dendogram was created by the neighbor-joining method based on the deduced amino acid sequence of GSTs. Bootstrap values are placed at the nodes. GenBank accession numbers are as follows: *Aedes aegypti* (Theta), Q16X19; *Homo sapiens* (Alpha), P08263; *Ixodes scapularis* A, XP_002401749.1; *Ixodes scapularis* B, XP_002434207.1; *Rattus norvegicus*, NP_803175.1; *Mus muculus,* NP_032209.1; *Haemaphysalis longicornis*, AAQ7444.1; *Dermacentor variabilis*, ACF35504.1; *Rhipicephalus* (*Boophilus*) *annulatus,* ABR24785.1; *Rhipicephalus sanguineus*, AGK29895.1; *Anopheles gambiae* (Delta), Q8MUS1; *Drosophila melanogaster*, (Epsilon) Q7KK90; *Homo sapiens*, (Zeta) O43708; and *Proteus mirabilis*, (Beta) P15214

A phylogenetic tree was also constructed using amino acid sequences of GSTs from different species to further analyze the identity of the GSTs (Fig. 2). HlGST was found to be closely related to the mu-class GSTs of *D. variabilis* and *I. scapularis* (GenBank: XP_002401749.1), while HlGST2 is closely related to *I. scapularis* GST (GenBank: XP_002434207.1). These results demonstrate that the newly identified HlGST2 also belongs to the mu-class of GST, due to its more than 40% similarity to the mammalian mu-class of GST [6] and the presence of the mu-loop (Additional file 3: Figure S3) [22].

### Expression of recombinant GSTs

**Fig. 3:**
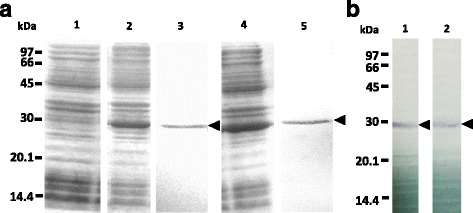
SDS-PAGE (**a**) and Western blotting (**b**) of recombinant GSTs. **a** The column farthest left includes markers of molecular weights. Lane 1: bacterial lysate of empty vector; Lane 2: bacterial lysate of HlGST after induction by 1 mM IPTG; Lane 3: bacterial lysate of HlGST after purification by HisTrap affinity chromatography; Lane 4: bacterial lysate of HlGST2 after induction by 1 mM IPTG; Lane 5: bacterial lysate of HlGST2 after purification by HisTrap affinity chromatography. The lysates were run on a 12% SDS-PAGE gel. Gels were stained using Coomassie Blue staining solution. **b** The column farthest left indicates molecular weight markers. Lane 1: recombinant HlGST; Lane 2: recombinant HlGST2. Bands were visualized using 5-Bromo-4-chloro-3-indolylphosphate/Nitroblue Tetrazolium (BCIP/NBT) Calbiochem® (Merck KGaA, Darmstadt, Germany). Arrowheads indicate the bands for recombinant HlGST and HlGST2 proteins

The expression of recombinant GSTs was performed using *E. coli* BL21 cells with pRSET A as the vector. The expression was induced by IPTG at a final concentration of 1 mM at 37 °C. After expression, purification by affinity chromatography was carried out. The eluted protein was checked by 12% SDS-PAGE and seen as single bands, which indicated purity. The purified recombinant HlGST molecular weight was approximately 28 kDa, while recombinant HlGST2 has an approximate molecular weight of 29 kDa. Recombinant GSTs contain fragments of the his-tag protein, which could account for the difference in the calculated molecular weights of 25.7 kDa and 26.3 kDa for HlGST and HlGST2, respectively (Fig. 3). Western blotting using an anti-histidine antibody showed positive signals, indicating the presence of histidine-tagged GSTs (Fig. 3). These results demonstrate the successful expression of histidine-tagged recombinant GST using the *E. coli* expression system.

### GST specific enzymatic activity

**Fig. 4:**
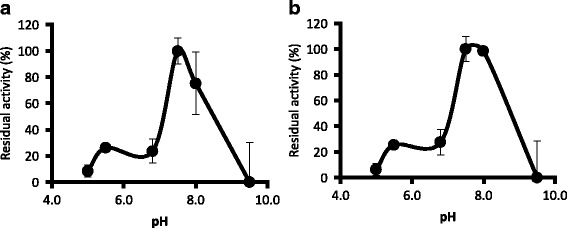
Effect of pH on the enzymatic activity of recombinant HlGST (**a**) and HlGST2 (**b**). Buffers were 0.1 M citrate for pH 5.0–5.5 and Tris-HCl for pH 6.5–8.5. Error bars represent the standard deviation of three replicates, and the remaining activities were recorded as percentages relative to the highest activity

The specific activity of recombinant HlGST and HlGST2 was determined through its ability to conjugate CDNB. CDNB has been used as a substrate in previous studies, and it has shown that mu-class GSTs react better with CDNB as compared with other substrates, such as 1,2-dichloro-4-nitrobenzene (DCNB) [15]. The enzymatic activity of recombinant GSTs toward CDNB is 9.75 ± 3.04 units/mg protein for recombinant HlGST and 11.63 ± 4.08 units/mg protein for recombinant HlGST2 (Table 2). Recombinant HlGST2 appears to have higher enzymatic activity than HlGST. To further characterize the GSTs, the optimum pH of the activity of recombinant GSTs was determined by checking its activity in buffer with different pHs. The pH at which the activity of both GSTs is the highest, or the optimum pH, is 7.5–8.0 (Fig. 4). These results demonstrate that the expressed recombinant GSTs could conjugate GSH with the substrate CDNB and possess almost the same rate of conjugation activity in a similar range of pH.

**Table 2:** Specific activity of each recombinant GST with the substrate CDNB. Values are presented as mean ± SD. The concentration of CDNB and GSH used are 1 mM and 5 mM, respectively. Each experiment was performed at least three times and each assay was run in triplicate

Enzyme	Enzyme activity (units/mg protein)
Equine liver GST^a^	11.01 ± 2.60
rHlPRX2^b^	nd
rHlGST^c^	9.24 ± 3.05
rHlGST2^d^	9.46 ± 1.97

Abbreviation: *nd* none detected

^a^Equine liver GST was used as a positive control

^b^Recombinant *H. longicornis* peroxiredoxin2 was used as the negative control

^c^Recombinant HlGST

^d^Recombinant HlGST2

### Enzyme kinetics of GSTs

To further establish the characteristics of these GSTs, we determined the enzyme kinetics constants of these GSTs, using the Michaelis-Menten equation and the Lineweaver-Burk plot. When the concentration of GSH is kept constant, HlGST has a *V*_*max*_ of 11.70 ± 1.92 units/mg protein and a *K*_*m*_ of 0.82 ± 0.14 mM, whereas HlGST2 has a *V*_*max*_ of 14.72 ± 0.56 units/mg protein and a *K*_*m*_ of 0.61 ± 0.20 mM. Meanwhile, when the concentration of CDNB is kept constant, HlGST has a *V*_*max*_ of 10.40 ± 1.77 units/mg protein and a *K*_*m*_ of 0.64 ± 0.32 mM, whereas HlGST2 has a *V*_*max*_ of 11.01 ± 0.21 units/mg protein and a *K*_*m*_ of 0.53 ± 0.19 mM (Table 3). These results demonstrate that recombinant GSTs have a high affinity toward GSH and the known GST substrate, CDNB. They also indicate a high ability of the recombinant GSTs to conjugate with CDNB and GSH.

**Table 3:** Recombinant GST kinetic constants. Values are presented as mean ± SD. Each experiment was performed at least three times and each assay was run in triplicate

Enzyme	CDNB kinetic constant^a^	GSH kinetic constant^b^
rHlGST^c^		
*V*_*max*_ (units/mg protein)	11.70 ± 1.92	10.40 ± 1.77
*K*_*m*_ (mM)	0.82 ± 0.14	0.64 ± 0.32
rHlGST2^d^		
*V*_*max*_ (units/mg protein)	14.72 ± 0.56	11.01 ± 0.21
*K*_*m*_ (mM)	0.61 ± 0.20	0.53 ± 0.19

^a^CDNB, the common substrate, is used in increasing concentration from 0.125–2 mM to determine the kinetic constants

^b^GSH is used in increasing concentration for 0.5–5 mM to determine the kinetic constants

^c^Recombinant HlGST

^d^Recombinant HlGST2

### Transcription profiles of *GSTs*

**Fig. 5:**
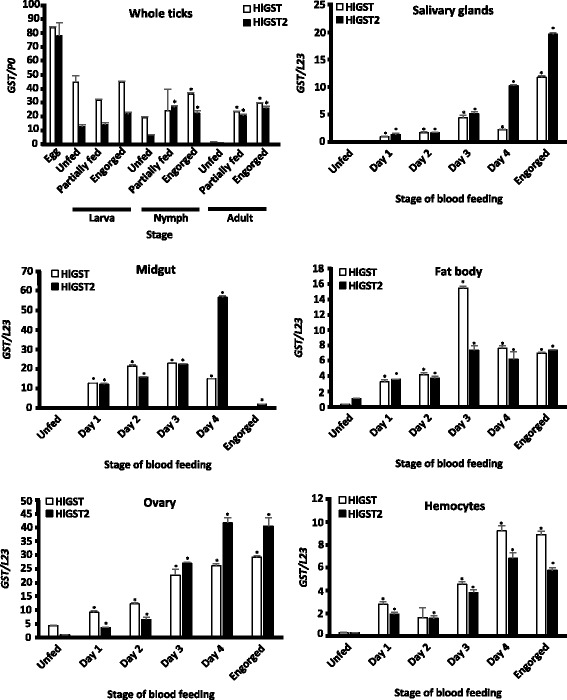
Transcription profiles of *GST* genes in different stages and tissues of ticks during blood-feeding. Total mRNA was prepared from different stages and tissues after dissection. *P0* and *L23* primers were used as controls for whole ticks and tissues, respectively. Error bar represents the mean ± standard deviation. ^*^P < 0.05, significantly different by Student's t*-*test as compared to the unfed at the same stage. *P*-values and *t-*values are indicated in Additional file 6: Table S1

The transcription profiles of *HlGST* and *HlGST2* genes from different developmental stages and organs of ticks during blood-feeding were checked using real-time PCR (Fig. 5). It was observed that both *HlGST* and *HlGST2* genes were constitutively expressed at all developmental stages, with increasing expression observed toward engorgement in the nymph and adult stages for *HlGST* and in all stages for *HlGST2* genes. Relatively strong expressions of both HlGST and HlGST2 genes were observed in the eggs. The gene transcription during the larval stages shows that *HlGST* appears to be maintained at a high transcription level. Both *HlGST* and *HlGST2* genes were also expressed in all organs. In different organs of female ticks, such as the salivary glands, ovaries, fat bodies, and hemocytes, gene expression increases as blood-feeding progresses, continuously increasing until engorgement. In the midgut, increasing transcription during the course of blood-feeding was also observed, except in the engorged stage, wherein there is a decreased expression of both *HlGST* and *HlGST2* genes. These results demonstrate that blood-feeding can trigger an upregulation of the transcription of *GST* genes. This fact could indicate the genes’ possible role in coping with oxidative stress caused by blood-feeding.

### Protein expression profiles of GSTs

**Fig. 6:**
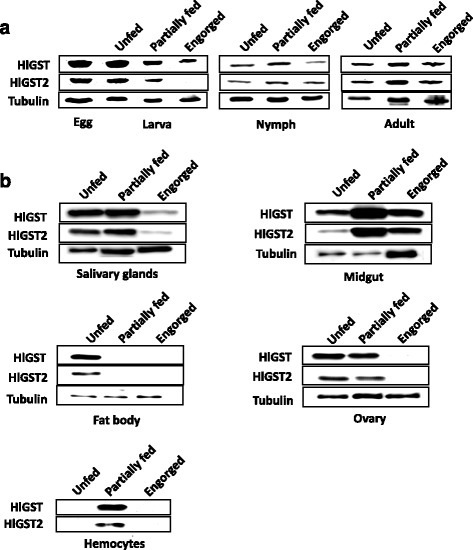
Expression profiles of GSTs in different tick stages (**a**) and organs (**b**) during blood-feeding. Proteins were prepared from different stages and tissues after dissection. Antiserum against tubulin was used as a control for Western blotting. Western blotting results are shown as representative data of three separate experiments showing the same trend. Since no band can be seen using the tubulin antisera on hemocytes, the protein concentration was determined using Micro BCA and maintained at 390 ng before loading for Western blotting

The protein expression of endogenous GSTs in different developmental stages and organs was determined through Western blotting analysis using specific anti-GST sera (Fig. 6 and Additional file 4: Figure S4). The GST protein expression during the larval stage has a tendency to decrease during the course of blood-feeding to engorgement, while in the nymph and adult stages, protein expression tends to increase during feeding and then decrease at the end of blood-feeding. In the midgut and hemocytes, GST expression increased during the partially fed state of blood-feeding; however, in other organs examined, such as the salivary glands, fat body, and ovaries, the expression of both HlGST and HlGST2 proteins decreased as blood-feeding progressed to engorgement. These results demonstrate that GST protein expression in ticks is upregulated during periods of increased oxidative stress, specifically on organs exposed to such oxidative stress, such as the midgut. This further confirms the anti-oxidant function of GSTs during blood-feeding.

### Localization of HlGST and HlGST2 in different organs using IFAT

**Fig. 7:**
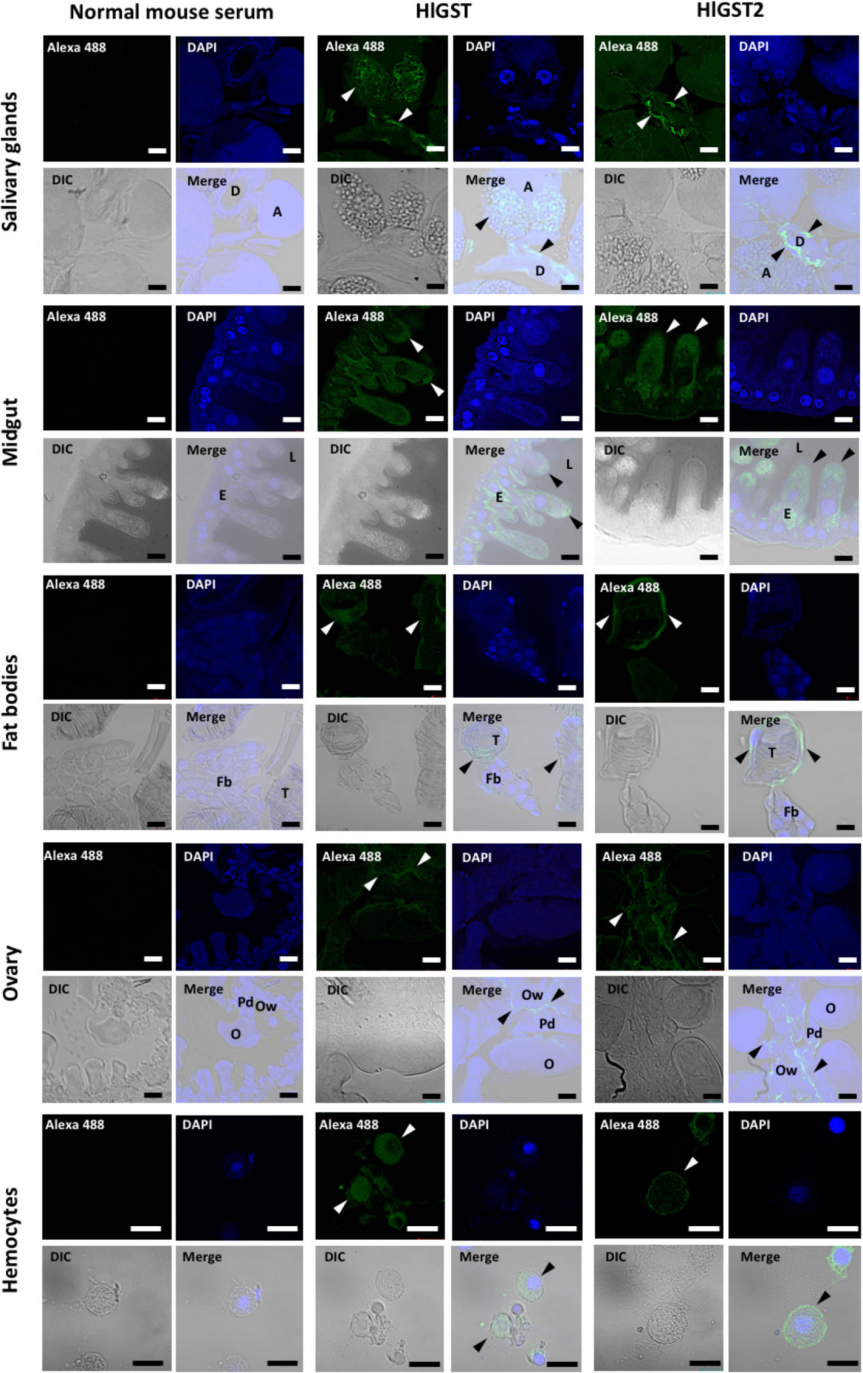
Localization of GSTs in tissues of partially fed adult ticks. Immunofluorescent antibody test (IFAT) was used to determine the localization of the GSTs in the different tissues of ticks. Antiserum against HlGST or HlGST2 was used for the primary antibody, anti-mouse IgG conjugated with Alexa 488 was used for the secondary antibody, and nuclei were visualized using DAPI. Normal mouse serum was used for a control. The tissues were visualized using confocal microscope. *Abbreviations*: Salivary glands (A, acinus; D, salivary ducts); Midgut (E, enterocytes; L, lumen; Fat bodies (T, tracheal complex; Fb, fat body cells); Ovary (O, oocyte; Pd, pedicel; Ow, ovarian wall). Arrows show positive GST fluorescence. *Scale-bars*: 20 μm

The localizations of endogenous HlGST and HlGST2 in partially fed adult salivary glands, midguts, fat bodies, ovaries, and hemocytes were demonstrated using IFAT (Fig. 7). Both HlGST and HlGST2 are found in the cytoplasm of cells. GSTs in the salivary glands are observed in the ducts and its epithelial cells of the non-degenerated acinus. A positive reaction was observed in the apical part of the epithelium of the midgut cells. In the fat body, a positive reaction was observed in the tracheal complex. In the ovaries, a positive reaction for GST was observed mainly in the pedicels and ovarian wall. Positive fluorescence in the cytoplasm of hemocytes was also observed. However, strong HlGST2 fluorescence was observed on the periphery of the hemocytes, while for HlGST, it was observed at scattered locations throughout.

**Fig. 8:**
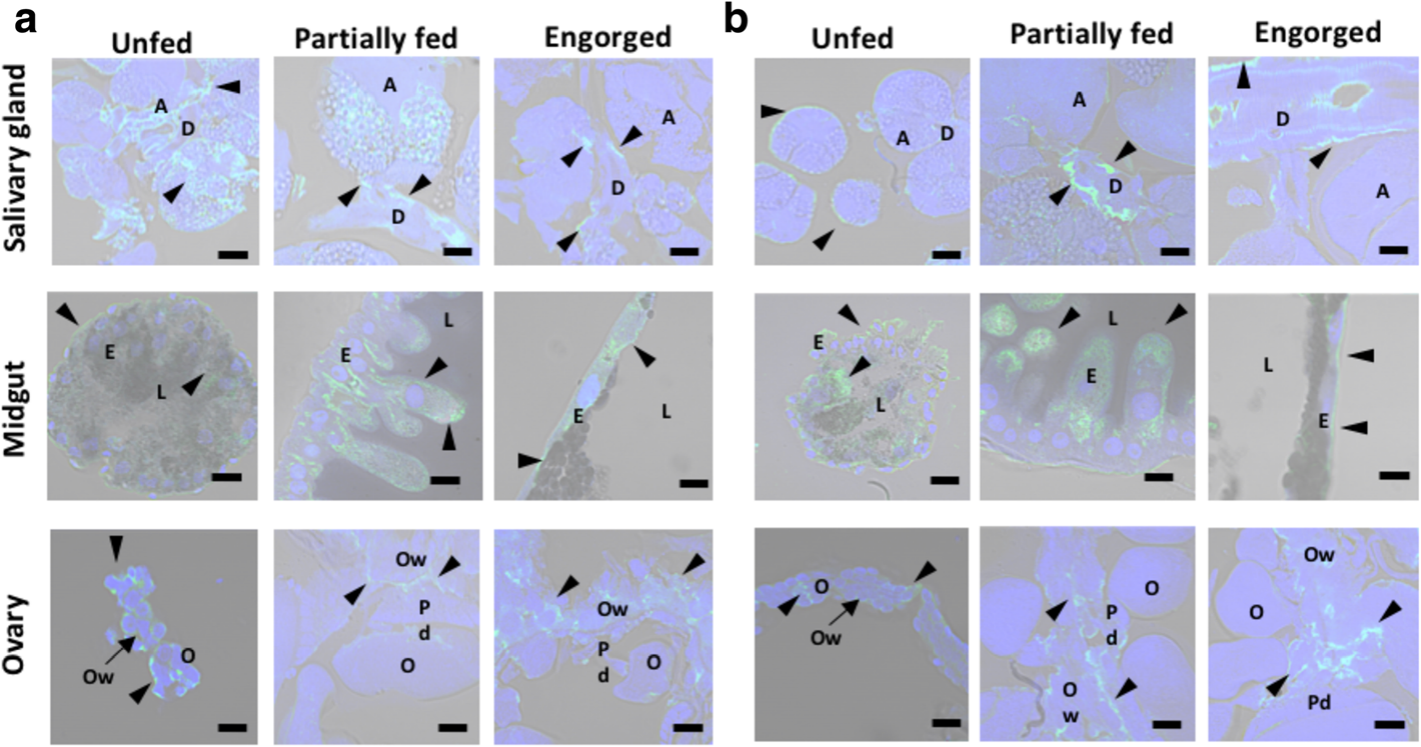
Examination of HlGST **(a**) and HlGST2 (**b**) in selected tissues during blood-feeding. The salivary glands, midgut, and ovary were observed during blood-feeding of adult ticks by indirect immunofluorescent antibody test (IFAT) using a confocal laser scanning microscope**.** Antiserum against HlGST or HlGST2 was used for the primary antibody, anti-mouse IgG conjugated with Alexa 488 was used for the secondary antibody, and nuclei were visualized using DAPI. Normal mouse serum was used for a control. *Abbreviations*: Salivary glands (A, acinus; D, salivary ducts); Midgut (E, enterocytes; L, lumen); Ovary (O, oocyte; Pd, pedicel; Ow, ovarian wall). Arrows show the positive fluorescence of GST. *Scale-bars*: 20 μm

IFAT was used to further characterize the role of GSTs in major organs such as salivary glands, midguts, and ovaries from female ticks at different blood-feeding stages (Fig. 8). In the salivary glands, GSTs are spread throughout the acinus; however, during blood-feeding, GSTs shift to being more expressed in the ducts during the partially fed and engorged stages. In the unfed midgut, fluorescence is also scattered throughout the digestive cells, then shifts toward the apical part of the epithelium in partially fed ticks and toward the basal membrane in the engorged stage. Fluorescence is limited in the ovarian wall and the pedicels of the ovary, but not in the oocytes, throughout the duration of blood-feeding. These results demonstrate that GST proteins tend to vary depending on the blood-feeding stage and eventually the levels of oxidative stress.

## Discussion

Multiple isoenzymes of GSTs have been observed in all eukaryotes [6]. *In silico* analysis of the *Ixodes scapularis* gene database showed 35 genes of GSTs, of which 14 belong to the mu-class GST [23]. In *Dermacentor variabilis* and *Rhipicephalus* (*Boophilus*) *annulatus*, multiple GSTs have also been found [14, 24]. The presence of multiple forms of GSTs could prove to be important for species to counter most, if not all, foreign or endogenous compounds that could affect them [15]. One GST has already been identified and partially characterized in *H. longicornis* [2]. Here, a novel GST from *H. longicornis*, HlGST2, was identified and characterized with the previously identified GST, HlGST.

The smaller predicted substrate binding site of HlGST2 located at the C-terminal domain could account for the faster activity rate of HlGST2 because of the possibly fewer enzyme-substrate interactions that could slow the rate of product release from the enzymes [25].

The effect of pH on enzymatic activity has been an important factor in determining the protein structure and function [15]. The optimal pH for activity toward CDNB of 7.5–8.0 is in the same range as that of *R.* (*B*.) *annulatus* GST. The optimum pH could vary depending on the compound used to test the GST activity, which could range from 6.5–9.5. If we consider only CDNB, the values also represent the modal tendency of several species at pH 7.5–8.0. Loss or decrease of enzymatic activity at lower or higher pH could be the result of the ionizing activity of both CDNB and GSH that occurs at pH 6–7 [15, 26]. This result could indicate stability and the capability of GSTs to function in conditions closer to intracellular conditions.

The *V*_*max*_ values for both recombinant GSTs are below those of the recombinant GST of *R.* (*B*.) *annulatus* (rBaGST), which are 75.2 units/mg protein for CDNB and 48.8 units/mg protein for GSH, it is important to consider that the presence of galactosidase in rBaGST or the histidine tag in *H. longicornis* recombinant GSTs could result in an altered level of activity of recombinant GSTs. The relatively low *K*_*m*_ values (< 1) of both GSTs for both GSH and CDNB may indicate a high rate of conjugation that could be attributed to the enzymes [22]. These *K*_*m*_ values are close to the *K*_*m*_ values for rBaGST, which are 0.57 mM and 0.79 mM for functions of CDNB and GSH, respectively [15]. The *K*_*m*_ values of GSH are also close to the values frequently cited in various literature (61%), which fall in a range of 0.19–0.79 mM [26]. Thus, it is reasonable to think that this newly identified HlGST2 of the hard tick, *H. longicornis*, could provide the tick with a coping mechanism for metabolizing xenobiotic and endogenous products. In addition, these enzyme kinetic parameters could be a basis for comparison for future inhibition studies, in which changes in these values could determine the type of inhibition if an inhibitor is added.

Upregulation of *GST* genes during blood-feeding suggest the importance of GSTs to oxidative stress management. It has also been assumed that reactive oxygen is a transduction signal that mediates the gene transcription of *GSTs* [27]. Thus, during blood-feeding, when the intracellular digestion of blood is high, high levels of reactive oxygen species are produced, which could induce the transcription of *GST* genes [14]. Upregulation brought about by blood-feeding was also observed in other blood-feeding arthropods, such as the *Aedes aegypti* [7], and in other ticks, such as *I. ricinus* and *R.* (*B.*) *microplus* [13, 28]. A high gene transcription profile is observed in eggs since they undergo rapid metabolism due to embryonic development, which could result in the production of an endogenous toxic substance in the eggs; thus, detoxification enzymes are important [5]. Interestingly, the downregulation of *GST* genes during blood-feeding was only observed in the engorged midgut. This could indicate that during this state, the amount of oxidative stress in midgut cells is reduced. The decreased expression could be a result of the nature of blood-feeding in ticks. At the start of blood-feeding, gut epithelia undergo a slow feeding phase, in which the tick prepares its midgut cells for blood intake. Here, some undifferentiated cells begin to differentiate and proliferate. This is also the period in which there is a rapid digestion of ingested cells. In effect, during this stage, ticks are more prone to oxidative stress brought about by rapid metabolism due to cell differentiation and proliferation, and the lysis of ingested cells that leads to the liberation of compounds may also trigger oxidative stress, such as heme aggregates. Afterward, ticks undergo a fast feeding period and eventually drop off the host. During this stage, the midgut would then act as a blood reservoir and the rate of intracellular digestion also becomes low [29]. Low intracellular digestion also results in low ROS emergence and low iron metabolism. The downregulation of mu-class *GST*s was also observed in engorged midgut cells of *I. ricinus.* [14]. Since GST proteins are said to be transcriptionally regulated [27], protein expression in the midgut also appeared to decrease during the engorged stage.

Western blotting analysis revealed decreasing expression in several organs, such as salivary glands, fat bodies, and ovaries, as compared with their increasing gene transcription profile. The decreased oxidative stress during the engorged stage, even with increased transcription in these organs, resulted in an expression level of GST proteins below the Western blotting’s capability to detect. The movement of heme, a potentially oxidant molecule, through its carrier protein throughout blood-feeding and, most especially, during the post attachment stage [30, 31], could trigger the upregulation of gene transcription in these organs.

In the salivary glands, the decrease in GST protein expression could also be due to GSTs being secreted through the saliva during the fully engorged stage, as previously observed in other tick species, such as *H. longicornis*, *I. scapularis*, and *Amblyomma americanum* [32–34]. IFAT results also demonstrated this possible release, as manifested by the apparent shift of GSTs from the acinus toward the salivary ducts during blood-feeding. The release of GST from its cytosolic localization has also been reported in mammalian organs such as plasma from the platelets, bile from the liver, and seminiferous tubule fluid from the testes. The release is said to be an energy-requiring active process rather than a secretory process [35–37]. The release could be a possible extracellular detoxification capability of GSTs. The degeneration process in the salivary glands during the end of blood-feeding could also be a factor in the decrease of protein expression, as it may cause the release of the GST protein. However, these are just proposed theories, and further studies are warranted to validate these claims. IFAT results also indicated an apparent movement of GSTs from whole cells in the unfed to the apical area of the epithelium in the partially fed to the basal cells in the engorged stage. This apparent movement of GSTs during blood-feeding could indicate the specific location in the midgut where there is exposure to exogenous substances in the blood meal. GST expression could then be an indicator of the amount and location of oxidative stress in the midgut. Among the organs, the midgut showed the highest gene and protein expression; its level of expression could determine the overall trend of GST protein in whole ticks as observed in nymphs and adults.

IFAT results of the fat bodies showed positive fluorescence on the tracheal complex of ticks. This could indicate the role of GST in the detoxification of xenobiotic compounds that may enter ticks through their spiracles. The spiracles and tracheal systems of insects were also observed to be points of entry for insecticides [38]. The presence of GSTs on the organs responsible for the exchange of gases was also observed in the gills of several marine species as well as human lungs [26, 39–41]. In hemocytes, differences in localization were observed. While it is scattered in HLGST, surface localization was observed in HlGST2. Surface localization in mu-class GSTs was also observed in goat sperm [42]. This could be a key feature that needs further study as to how GSTs could play a role in the defense mechanism of the tick’s hemocytes and its role in tick immunity.

IFAT of the ovaries showed positive fluorescence in the ovarian wall and into the pedicels. This could be an indication of the mechanism protecting the ovaries of ticks from environmental chemicals that may be acquired through the genital aperture, which is connected to the ovary *via* the oviduct [26, 43]. Also, signals in the pedicels of the ovary could reflect its role in the development of oocytes. The presence of numerous mitochondria in the pedicels [44] indicates that they have very high activity, thus consuming lots of oxygen for ATP production. Therefore, pedicels are very metabolically active cells that generate ROS, which in turn could result in the expression of GSTs.

The possibility of the HlGST and HlGST2 antisera to cross react with other GSTs of *H. longicornis* in Western blotting and IFAT remains uncertain since anti-GST sera are known to cross react with other GST proteins with high homology [2, 45]. Nevertheless, the similarity in the trend in expression and localization indicate how at least HlGST and HlGST2, if not all mu-class GSTs of *H. longicornis*, responds to the oxidative stress.

The expression and localization of GSTs in different organs may indicate how ticks use GSTs as protective mechanism to cope with oxidative stress that may vary according to the specific needs of each organ for the enzyme, either as a carrier protein or as being metabolically active [9].

To further investigate the importance of GST and its effects on tick survival during blood-feeding and on reproductive parameters such as engorgement weight, egg weight, and hatching rate, a gene knockdown experiment was performed; however, preliminary results of the gene knockdown experiment (Additional file 5: Table S2) showed no significant differences in the above-mentioned parameters between the *GST*-knockdown group and the *EGFP*-knockdown group. In *R. microplus*, no significant differences were observed in terms of tick mortality and oviposition when *GST* is knocked down [46]. The probable reason is that GSTs could provide functional compensation between members of each family [47], although further studies must be conducted to prove this hypothesis. Vaccination experiments using the HLGST as the antigen, on the other hand, showed a decrease in the engorgement rate of ticks [45]. This could further establish the role of the GSTs during blood-feeding and reproduction, although the exact mechanism should be a subject of future studies.

## Conclusions

In summary, a new GST in the hard tick *H. longicornis* was identified, and recombinant GSTs were synthesized*.* Both GSTs of *H. longicornis* were characterized *in silico*, using various available software applications; in vitro, through studying the enzymatic activity and kinetics; and in vivo, through its gene and protein expression in whole ticks and different organs during blood-feeding and organ localization. The results have shown a positive correlation between the degree and localization of the GSTs with the degree and localization of oxidative stress occurring within the tick during blood-feeding. This close relationship could indicate that GSTs play a possible role in coping with oxidative stress brought about by blood-feeding. The information gathered here could be a tool for designing tick control methods, such as the proper timing of applying acaricides. Since GSTs have also been a target in the design of anti-tick vaccines, the identification of yet another GST that has a lower homology is of importance in better vaccine design. Also, some discrepancies between transcription and expression profiles were found. These results emphasize the notion that GST studies should involve the gene, protein, and enzyme activity; it should also be a multi-faceted study that takes into consideration the capability of this ubiquitous enzyme.

## Additional files

Additional file 1: Figure S1. RT-PCR (a) and Western blotting (b) of knockdown ticks. Ticks were silenced by injecting 1 μg of double-stranded RNA (dsRNA) per tick. a Total RNA was extracted from whole 4-day fed *GST* and *EGFP* knockdown ticks. cDNA was synthesized and subjected to RT-PCR. PCR products were run on 1.5% TAE agarose gel and stained with ethidium bromide. *Actin* was used as loading control. b Protein lysates were extracted from whole 4-day-fed *HlGST, HlGST*2*, HlGST1/2,* and *EGFP* knockdown ticks. Protein lysates were run on 12% SDS-PAGE gel before being transferred to polyvinylidene difluoride (PVDF) membranes and subjected to Western blotting. Mouse tubulin antiserum was used as control. (PDF 200 kb)

Additional file 2: Figure S2. Nucleotide and deduced amino acid sequences of HlGST (a) and HlGST2 (b) of *Haemaphysalis longicornis.* Start and stop codons are underlined. Predicted glutathione and substrate binding sites are shaded in black and gray, respectively. The putative polyadenylation signal, AATAAA, is double underlined. (PDF 53 kb)

Additional file 3: Figure S3. The modeled tertiary structures of HlGST (a) and HlGST2 (b). The model is based on template c1b8xA [48] constructed using PHYRE2 software [49]. Green indicates the N-terminal domain containing the GSH binding site (orange), while red indicates the C-terminal domain containing the substrate binding site (blue). The mu-loop is indicated by bluish green. (PDF 193 kb)

Additional file 4: Figure S4. Western blotting of adult female ticks during blood-feeding. Protein lysates were extracted from adult female ticks during the different stages of blood-feeding. Protein lysates were run on 12% SDS-PAGE gel before being transferred to PVDF membranes and subjected to Western blotting. Mouse tubulin antiserum was used as control. Leftmost lane indicates markers for molecular weight. (PDF 253 kb)

Additional file 5: Table S2. Effect of *HlGST* and/or *HlGST*2 knockdown on ticks. (DOCX 36 kb)

Additional file 6: Table S1. *P-* and *t*-values of *GST* transcription profiles. (DOCX 40 kb)

## Abbreviations

CDNB: 1-chloro-2,4-dinitrobenzene
DCNB: 1,2-dichloro-4-nitrobenzene
dsRNA: double stranded RNA
EGFP: enhanced green fluorescent protein
EST: expressed sequence tags
GSH: glutathione
GST: glutathione S-transferase
HlGST: GST of *H. longicornis*
IFAT: immunofluorescent antibody test
IPTG: isopropyl β-D-1-thiogalactiopyranoside
LB: Luria-Bertani
ORF: open reading frame
PBS: phosphate buffered saline
pI: isoelectric point
PVDF: polyvinylidene difluoride
rBaGST: recombinant GST of *R.*(*B*.) *annulatus*
ROS: reactive oxygen species
SDS-PAGE: sodium dodecyl sulphate polyacrylamide gel electrophoresis
