# Supplementary figures and images for: Characterization and expression analysis of a newly identified glutathione S-transferase of the hard tick Haemaphysalis longicornis during blood-feeding
Source: Parasit Vectors. 2018 Feb 8;11:91. doi: 10.1186/s13071-018-2667-1 (PMC5806375; doi:10.1186/s13071-018-2667-1)

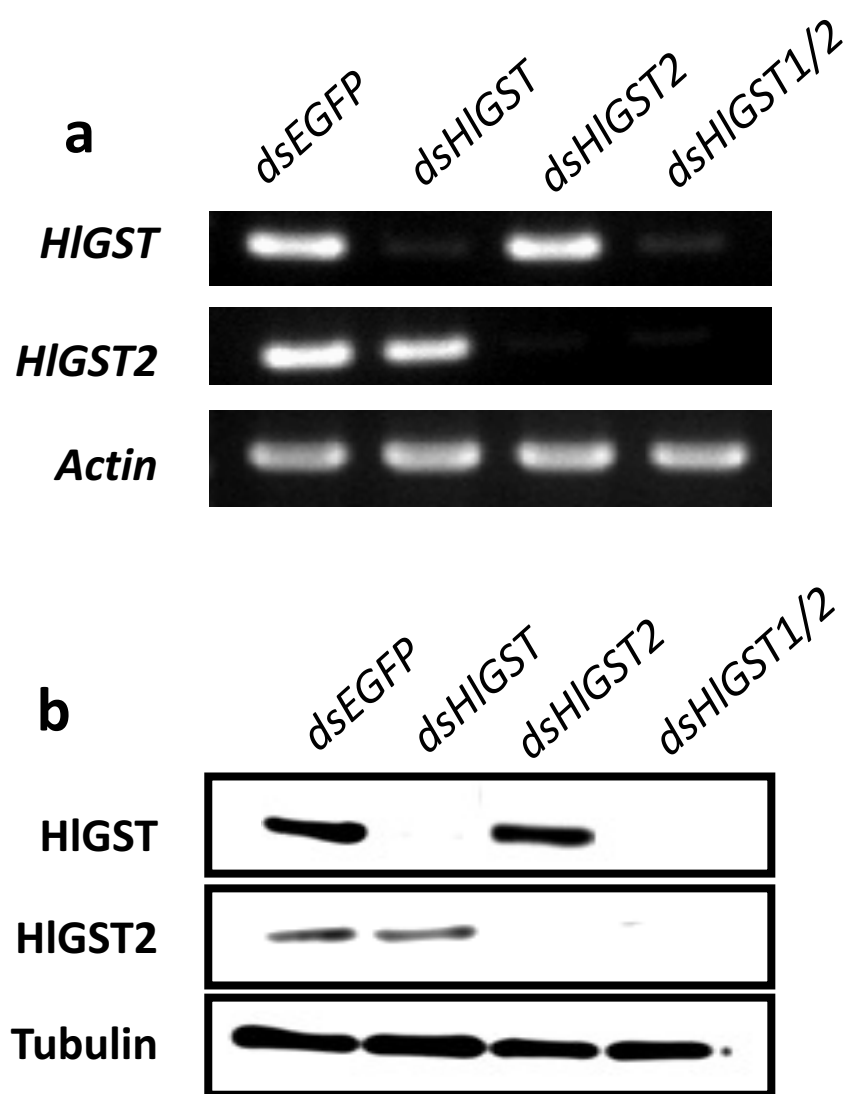

Supplement: Supplementary file 1 — RT-PCR (a) and Western blotting (b) of knockdown ticks. Ticks were silenced by injecting 1 μg of double-stranded RNA (dsRNA) per tick. a Total RNA was extracted from whole 4-day fed GST and EGFP knockdown ticks. cDNA was synthesized and subjected to RT-PCR. PCR products were run on 1.5% TAE agarose gel and stained with ethidium bromide. Actin was used as loading control. b Protein lysates were extracted from whole 4-day-fed HlGST, HlGST2, HlGST1/2, and EGFP knockdown ticks. Protein lysates were run on 12% SDS-PAGE gel before being transferred to polyvinylidene difluoride (PVDF) membranes and subjected to Western blotting. Mouse tubulin antiserum was used as control. (PDF 200 kb) [file 13071_2018_2667_MOESM1_ESM.pdf]

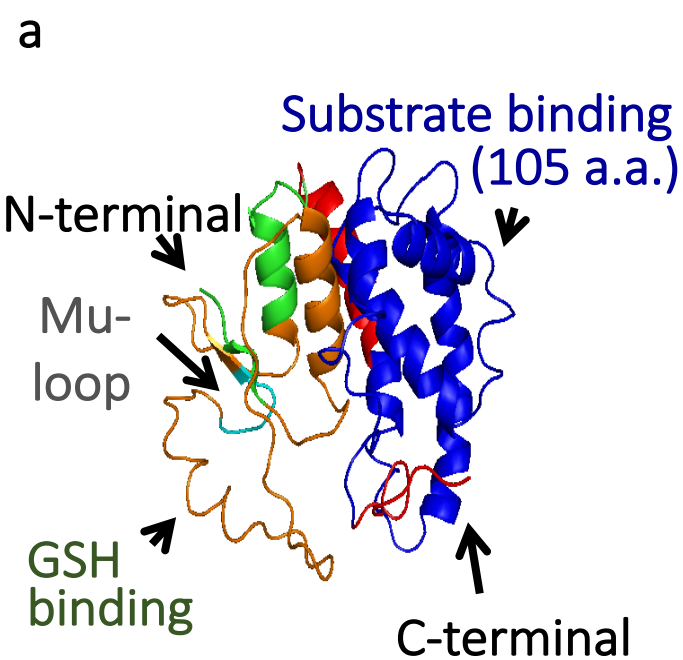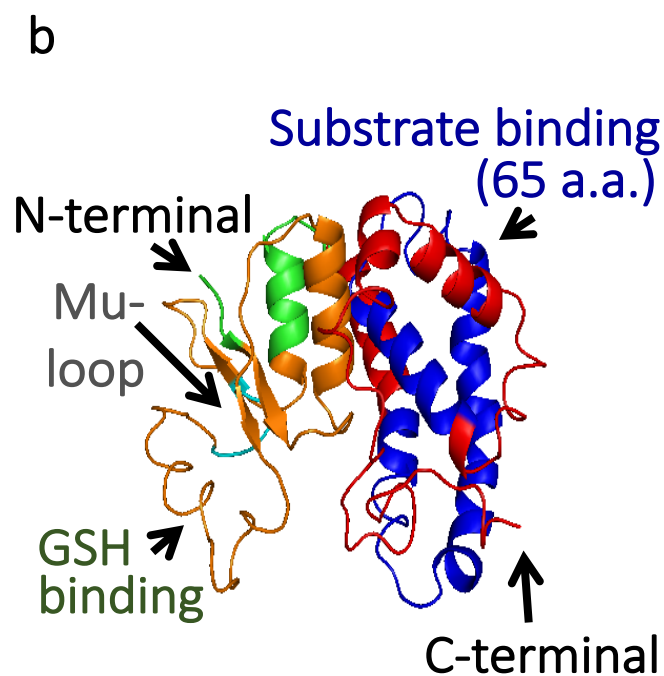

Supplement: Supplementary file 3 — The modeled tertiary structures of HlGST (a) and HlGST2 (b). The model is based on template c1b8xA [48] constructed using PHYRE2 software [49]. Green indicates the N-terminal domain containing the GSH binding site (orange), while red indicates the C-terminal domain containing the substrate binding site (blue). The mu-loop is indicated by bluish green. (PDF 193 kb) [file 13071_2018_2667_MOESM3_ESM.pdf]

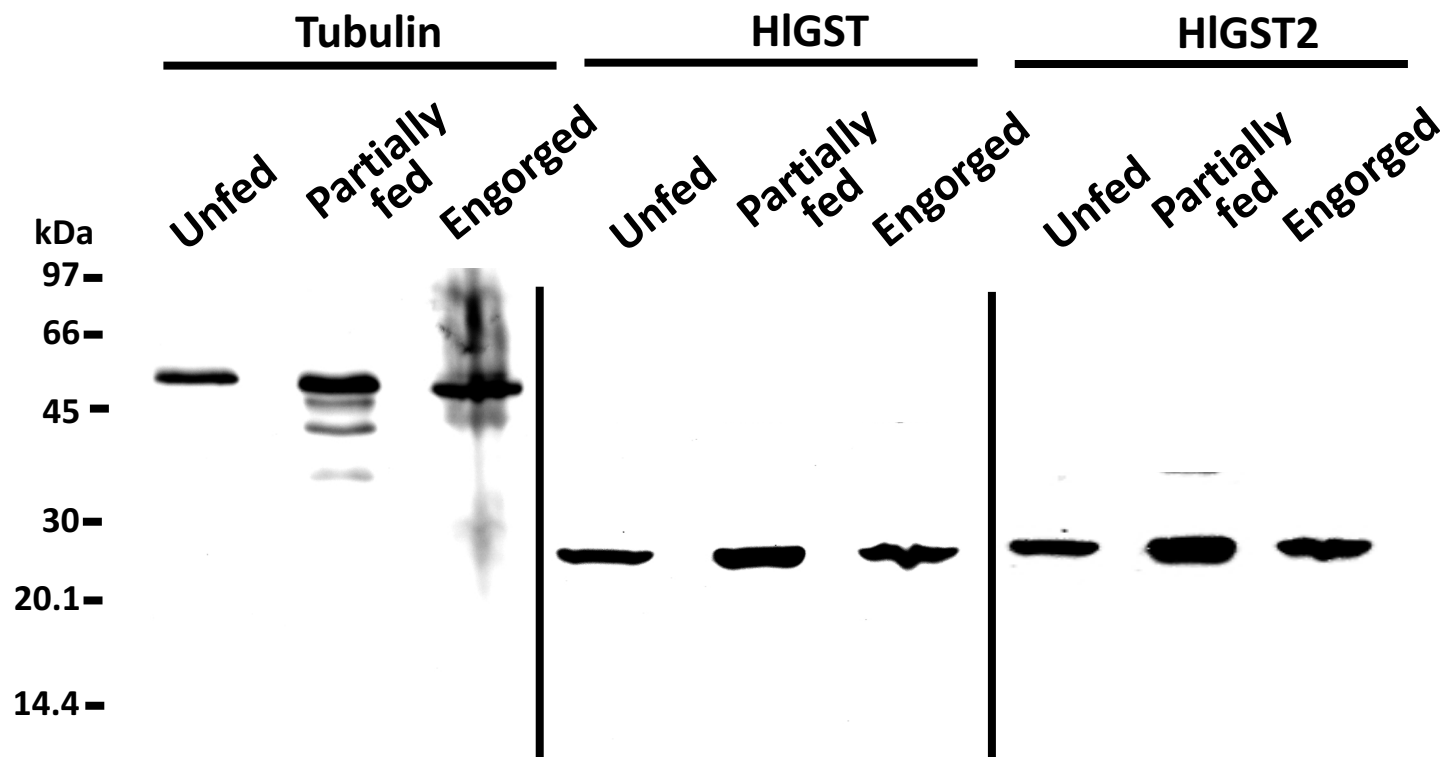

Supplement: Supplementary file 4 — Western blotting of adult female ticks during blood-feeding. Protein lysates were extracted from adult female ticks during the different stages of blood-feeding. Protein lysates were run on 12% SDS-PAGE gel before being transferred to PVDF membranes and subjected to Western blotting. Mouse tubulin antiserum was used as control. Leftmost lane indicates markers for molecular weight. (PDF 253 kb) [file 13071_2018_2667_MOESM4_ESM.pdf]
