# Supplementary material for: Characterization and expression analysis of a newly identified glutathione S-transferase of the hard tick Haemaphysalis longicornis during blood-feeding
Source: Parasit Vectors. 2018 Feb 8;11:91. doi: 10.1186/s13071-018-2667-1 (PMC5806375; doi:10.1186/s13071-018-2667-1)
Supplement: Supplementary file 2 — Nucleotide and deduced amino acid sequences of HlGST (a) and HlGST2 (b) of Haemaphysalis longicornis. Start and stop codons are underlined. Predicted glutathione and substrate binding sites are shaded in black and gray, respectively. The putative polyadenylation signal, AATAAA, is double underlined. (PDF 53 kb) [file 13071_2018_2667_MOESM2_ESM.pdf]

a

1 GATTCGCTTCTTGGATCTTGGCGGGTGTTGTGAACAGTCGCTGCATTTTCAACTGCTTTA

61 ACCATGGCTCCTATTCTCGGCTACTGGGACATTCGTGGACTGGCACAGCCAATCCGCCTG

M A P I L G Y W D I R G L A Q P I R L 19

121 CTGCTTGCCCACGCTGACGTCAAGGTGGAGGACAAGCGCTACTCATGCGGACCCCCTCCG

L L A H A D V K V E D K R Y S C G P P P 39

181 GATTTTGACCGCAGCGCCTGGCTCAAGGAGAAACACACCCTGGGCCTGGAGTTCCCCAAC

D F D R S A W L K E K H T L G L E F P N 59

241 CTGCCTTACTACATTGATGGGGACGTGAAGCTCACCCAGAGCATGGCTATTCTGCGCTAC

L P Y Y I D G D V K L T Q S M A I L R Y 79

301 CTTGCCCGCAAGCATGGACTGGATGGCAAGACAGAGGCCGAAAAGCAACGGGTCGACGTC

L A R K H G L D G K T E A E K Q R V D V 99

361 ACGGAGCAGCAGTTTGCCGACTTCCGCATGAACTGGGTTCGCATGTGCTACAACCCAGAC

T E Q Q F A D F R M N W V R M C Y N P D 119

421 TTTGACAAGCTCAAGGTCGACTACCTCAAGAACTTGCCAGATGCGCTGAAGAGCTTCTCA

F D K L K V D Y L K N L P D A L K S F S 139

481 GAGTACCTTGGGAAGCACAAGTTCTTCGCTGGCGACCATGTCACCTACGTTGACTTCATC

E Y L G K H K F F A G D H V T Y V D F I 159

541 GCTTACGAGATGCTGGCTCAGCACCTCCTCTTTGCTCCGGACTGCCTGAAGGATTTCCCC

A Y E M L A Q H L L F A P D C L K D F P 179

601 AACCTGAAGGCCTTTGTGGACCGCGTTGAGGCTCTCCCCCACGTGGCGGCCTACCTGAAG

N L K A F V D R V E A L P H V A A Y L K 199

661 TCTGACAAGTGCATCAGCTGGCCCCTCAACGGCGACATGGCTAGCTTCGGCAGCAGGCTG

S D K C I S W P L N G D M A S F G S R L 219

721 CAGAAGAAGCCGTGAACAGCACTTCATACCCACTGTCCGCTTTGGCGTTTGCGTTCCCCC

Q K K P *

781 CAATAAAGTTTTTCCGGTGGTGCAGGTCC

b

1 GAGTTGTGCTCGATCAAGCTACCATGGCCCCTGTGCTGGGATACTGGGACATCCGAGGCC

M A P V L G Y W D I R G L 13

61 TTTGCGAGCCCATTCGCTACCTTCTGGCGCACGCTAAAGTCTCCTACGAGGATAAAAGGT

C E P I R Y L L A H A K V S Y E D K R Y 33

121 ACGGCTTCGGCAATGGTCCCGAACCCAGCCGCGACGAGTGGTTGGCCGACAAGTACAAGT

G F G N G P E P S R D E W L A D K Y K L 53

181 TGGGTCTGGACTTCCCCAACGTGCCGTACTACATCGACGGCGACGTCAAGCTGACGCAGA

G L D F P N V P Y Y I D G D V K L T Q S 73

241 GCATGGCCATCCTGCAGTACCTCGGCCGCAAGCACGGACTCGCCCCCAAGGACGAGGCCA

M A I L Q Y L G R K H G L A P K D E A T 93

301 CTCAGCTCCGCGTCGACGTGCTCCAGCTCACGGCGTTCGACGTGATCATGTGGGCAGTGC

Q L R V D V L Q L T A F D V I M W A V R 113

361 GCGTCTGCTACGACCCCGAGTACACCGAGGAGAAGCGGAAGCAGTTCCTGGTCGACGTGG

V C Y D P E Y T E E K R K Q F L V D V A 133

421 CCGACAAGCTGAAGCAGTTTGACTCGTACCTCTCCAAGTATGGTCCTTTCGGCGCCGGCA

D K L K Q F D S Y L S K Y G P F G A G K 153

481 AGTCAGCCACTTACGTCGACTTCTTGCTCTACGAGGCTCTCCAGATCGTGAAAATTCTTG

S A T Y V D F L L Y E A L Q I V K I L G 173

541 GCCCAAGCACGTTCCGCAAGGGCTACCCTCAGCTCGAGGAGTACTGCCAGCGCGTTGCTG

P S T F R K G Y P Q L E E Y C Q R V A A 193

601 CCCTTCCGGGAATGAAGGAGTATCTGGCCTCGGATCGCTTCAAGGCTTGGCCCATCTGGA

L P G M K E Y L A S D R F K A W P I W S 213

661 GCCCGTACGCAAAGGCGCTGGCGGCGCAGCACAAGCCGCCCGCTGACGACTGCTGAGCGA

P Y A K A L A A Q H K P P A D D C * 230

721 TCCCCGCCGGCCATCGCGCGTCGCAAGCGTTGACCGCAGTTCTAGTTTTTTGATGTTGTT

781 AATCTGGGATGCGACGCTATATACGGCTGTTACATTTTTCTTTTCAATAAACGGTTGCGT

841 CGTTTTGAGCGCTCTTCCGACAAAAAAAAA

Hernandez et al. Fig. S2
