# Supplementary material for: Characterization and expression analysis of a newly identified glutathione S-transferase of the hard tick Haemaphysalis longicornis during blood-feeding
Source: Parasit Vectors. 2018 Feb 8;11:91. doi: 10.1186/s13071-018-2667-1 (PMC5806375; doi:10.1186/s13071-018-2667-1)
Supplement: Supplementary file 5 — Effect of HlGST and/or HlGST2 knockdown on ticks. (DOCX 36 kb) [file 13071_2018_2667_MOESM5_ESM.docx]

**Table S2. Effect of *HlGST* and/or *HlGST2* knockdown on ticks**

| **Groups** | **Engorgement rate of ticks**  **(%)** | | **Engorged body weight of ticks**  **(mg)** | **Percentage of ticks that laid eggs**  **(%)** | **Egg weight (mg)** | **Hatching rate**  **(%)** |
| --- | --- | --- | --- | --- | --- | --- |
| **dsEGFP** | | 83.33  (25/30) | 92.3±30.5 | 84.00  (21/25) | 43.4±21.0 | 85.71  (18/21) |
| **dsHLGST** | | 86.67  (26/30) | 116.2±29.2 | 100.00  (26/26) | 51.6±20.4 | 84.62  (22/26) |
| **dsHlGST2** | | 83.33  (25/30) | 129.6±31.6 | 92.00  (23/25) | 60.0±18.9 | 91.30  (21/23) |
| **dsHLGST1/2** | | 93.33  (28/30) | 130.46±36.1 | 92.86  (26/28) | 59.8±21.0 | 61.54  (16/26) |
