# Supplementary material for: Characterization and expression analysis of a newly identified glutathione S-transferase of the hard tick Haemaphysalis longicornis during blood-feeding
Source: Parasit Vectors. 2018 Feb 8;11:91. doi: 10.1186/s13071-018-2667-1 (PMC5806375; doi:10.1186/s13071-018-2667-1)
Supplement: Supplementary file 6 — P- and t-values of GST transcription profiles. (DOCX 40 kb) [file 13071_2018_2667_MOESM6_ESM.docx]

**Table S1. *P* and *t* values of *GST* transcription profiles**

|  | *HlGST* | | *HlGST2* | |
| --- | --- | --- | --- | --- |
|  | *P* value | t_2_ value | *P* value | t_2_ value |
| **Developmental Stages** |  |  |  |  |
| **vs. Unfed larva** |  |  |  |  |
| Partially fed larva | 0.06194 | 3.82903 | 0.39572 | 1.07256 |
| Engorged larva | 0.97834 | 0.03063 | 0.01521 | 8.01694 |
| **vs. Unfed nymph** |  |  |  |  |
| Partially fed nymph | 0.69723 | 0.44927 | 0.00173 | 23.98273 |
| Engorged nymph | 0.00274 | 19.04997 | 0.00658 | 12.26419 |
| **vs. Unfed adult** |  |  |  |  |
| Partially fed adult | 0.00037 | 52.30793 | 0.00164 | 24.67052 |
| Engorged adult | 0.0000002 | 2088.77167 | 0.00125 | 28.20529 |
|  |  |  |  |  |
| **Organs** |  |  |  |  |
| **vs. Unfed salivary glands** | |  |  |  |
| Day 1 salivary glands | 0.00610 | 12.75020 | 0.02858 | 5.78775 |
| Day 2 salivary glands | 0.00167 | 24.44331 | 0.00625 | 12.58812 |
| Day 3 salivary glands | 0.00485 | 14.30696 | 0.00156 | 25.25885 |
| Day 4 salivary glands | 0.00108 | 30.41432 | 0.00029 | 58.40088 |
| Engorged salivary glands | 0.00046 | 46.44049 | 0.00009 | 102.60782 |
| **vs. Unfed midgut** |  |  |  |  |
| Day 1 midgut | 0.00016 | 79.78968 | 0.00191 | 22.84507 |
| Day 2 midgut | 0.00059 | 41.06029 | 0.00013 | 86.20447 |
| Day 3 midgut | 0.00021 | 69.12350 | 0.00012 | 91.32402 |
| Day 4 midgut | 0.00001 | 305.30899 | 0.00008 | 112.07056 |
| Engorged midgut | 0.13376 | 2.45192 | 0.00012 | 91.44430 |
| **vs. Unfed fat body** |  |  |  |  |
| Day 1 fat body | 0.00401 | 15.73427 | 0.00027 | 60.34285 |
| Day 2 fat body | 0.00243 | 20.25413 | 0.00389 | 15.98116 |
| Day 3 fat body | 0.00015 | 81.40836 | 0.00389 | 15.98052 |
| Day 4 fat body | 0.00098 | 31.85352 | 0.01674 | 7.63170 |
| Engorged fat body | 0.00023 | 65.26109 | 0.00016 | 79.62860 |
| **vs. Unfed ovary** |  |  |  |  |
| Day 1 ovary | 0.00499 | 14.09945 | 0.00201 | 22.25290777 |
| Day 2 ovary | 0.00188 | 23.01318 | 0.01060 | 9.635881114 |
| Day 3 ovary | 0.00663 | 12.22476 | 0.00004 | 154.206119 |
| Day 4 ovary | 0.00062 | 40.17655 | 0.00101 | 31.47916513 |
| Engorged ovary | 0.00023 | 65.97902 | 0.00300 | 18.21830748 |
| **vs. Unfed hemocytes** |  |  |  |  |
| Day 1 hemocytes | 0.00430 | 15.20552 | 0.00604 | 12.81343 |
| Day 2 hemocytes | 0.17761 | 2.04429 | 0.01899 | 7.15253 |
| Day 3 hemocytes | 0.00111 | 29.94812 | 0.00215 | 21.51952 |
| Day 4 hemocytes | 0.00141 | 26.57520 | 0.00255 | 19.77090 |
| Engorged hemocytes | 0.00066 | 38.76068 | 0.00100 | 31.52768 |
